# RNA binding protein NKAP protects glioblastoma cells from ferroptosis by promoting SLC7A11 mRNA splicing in an m^6^A-dependent manner

**DOI:** 10.1038/s41419-022-04524-2

**Published:** 2022-01-21

**Authors:** Shicheng Sun, Taihong Gao, Bo Pang, Xiangsheng Su, Changfa Guo, Rui Zhang, Qi Pang

**Affiliations:** 1 Department of Neurosurgery, Shandong Provincial Hospital, Cheeloo College of Medicine, Shandong University, Jinan, Shandong 250012 China; 2 Department of Neurosurgery, Qilu Hospital, Cheeloo College of Medicine, Shandong University, Jinan, 250012 Shandong China

**Keywords:** CNS cancer, Necroptosis, RNA modification, RNA splicing

## Abstract

Ferroptosis is a form of cell death characterized by lipid peroxidation. Previous studies have reported that knockout of NF-κB activating protein (NKAP), an RNA-binding protein, increased lipid peroxidation level in naive T cells and induced cell death in colon cancer cells. However, there was no literature reported the relationship between NKAP and ferroptosis in glioblastoma cells. Notably, the mechanism of NKAP modulating ferroptosis is still unknown. Here, we found NKAP knockdown induced cell death in glioblastoma cells. Silencing NKAP increased the cell sensitivity to ferroptosis inducers both in vitro and in vivo. Exogenous overexpression of NKAP promoted cell resistance to ferroptosis inducers by positively regulating a ferroptosis defense protein, namely cystine/glutamate antiporter (SLC7A11). The regulation of SLC7A11 by NKAP can be weakened by the m^6^A methylation inhibitor cycloleucine and knockdown of the m^6^A writer METTL3. NKAP combined the “RGAC” motif which was exactly in line with the m^6^A motif “RGACH” (R = A/G, H = A/U/C) uncovered by the m^6^A-sequence. RNA Immunoprecipitation (RIP) and Co-Immunoprecipitation (Co-IP) proved the interaction between NKAP and m^6^A on SLC7A11 transcript. Following its binding to m^6^A, NKAP recruited the splicing factor proline and glutamine-rich (SFPQ) to recognize the splice site and then conducted transcription termination site (TTS) splicing event on SLC7A11 transcript and the retention of the last exon, screened by RNA-sequence and Mass Spectrometry (MS). In conclusion, NKAP acted as a new ferroptosis suppressor by binding to m^6^A and then promoting SLC7A11 mRNA splicing and maturation.

## Background

Glioblastoma (grade IV glioma) is the most malignant and common primary brain tumor in adults [1, 2]. The standard therapy for newly diagnosed glioblastoma is radiotherapy with concurrent temozolomide (TMZ) following maximal resection of the tumor, with a 5-year overall relative survival rate of only 6.8% [3]. Targeted molecular therapy for glioblastoma showed a restricted curative effect [4]. Therefore, identifying new therapeutic targets with high sensitivity and specificity has always been the most important research direction for studies on glioblastoma treatment.

Ferroptosis was first identified as an iron-dependent form of cell death in 2012, which was characterized by lipid peroxidation at the molecular level and shrunken mitochondria at the ultrastructural level [5]. The chemical basis of ferroptosis can be roughly divided into three categories: oxidation of polyunsaturated fatty acids (PUFAs)-containing membrane phospholipids by reactive oxygen species (ROS); availability of redox-active iron; and antioxidant activity of the cytomembrane against lipid hydroperoxide [6]. The endogenous antioxidant activity of the cytomembrane against lipid hydroperoxide can be categorized into three systems: SLC7A11/glutathione (GSH)/phospholipid hydroperoxide (PLOOH)-reducing enzyme glutathione peroxidase 4 (GPX4), ferroptosis suppressor protein 1 (FSP1)/NAD(P)H/ubiquinol, and dihydroorotate dehydrogenase (DHODH)/ubiquinol [7–13]. System x_c_^−^, whose main component is SLC7A11, acts as a cystine/glutamate antiporter to synthesize GSH, which converts to oxidized glutathione (GSSG) under the catalytic action of GPX4 to neutralize the oxidative substances in the cell membrane [9–11]. Erastin and sulfasalazine (SAS) can interfere with cystine uptake by inhibiting system x_c_^−^ to induce ferroptosis [14]. iFSP1, a potent FSP1 inhibitor, was reported to induce ferroptosis in GPX4 knockout cells [13]. Cancer therapies underlying ferroptosis attracted much attention with high expectations in recent years [15]. Ferroptosis-inducing drugs were found to exert a positive effect on glioblastoma therapies, which not only killed tumors directly [16] but also improved the sensitivity to radiotherapy and chemotherapy [17, 18]. However, glioblastomas could develop drug resistance [16], which restricted the application and development of ferroptosis-related therapies. So, exploring intervention measures for ferroptosis resistance had considerable potential clinical value.

NKAP is an evolutionarily conserved nuclear protein [19] and is involved in many physiological processes and diseases. NKAP acts as an RNA-binding protein to play a role in RNA splicing and processing [20, 21]. NKAP is considered a key regulator of mitosis based on its participation in chromosome alignment, and thus, might contribute to tumorigenesis [22]. NKAP also functions as a transcriptional repressor of the Notch target genes by associating with the Notch corepressor complex [23]. Recently, a study reported that NKAP acts as a reader of m^6^A during miRNA processing and maturation to promote pancreatic cancer progression [24]. NKAP functions as an oncogene and participates in the progression of cancer, including gastric cancer [25], colon cancer [26], and renal cell carcinoma [27]. In recent years, research groups from Mayo Clinic and the University of Pennsylvania reported that NKAP played a vital role in immune cell maturation and maintenance [28–33]. They found that deletion of NKAP in Treg cells and hematopoietic stem cells leads to cell death [28–30]. An interesting aspect that attracted us most was that NKAP knockdown induced increased lipid peroxidation in naive T cells [31] and a higher death rate in colon cancer cells [26]; these findings were similar to some of our research results on glioblastoma. In 2019, we reported that NKAP alters tumor immune microenvironment and promotes glioma growth. However, it remained unclear why NKAP deletion caused glioblastoma cell death [34].

In the present study, we demonstrated that NKAP knockdown induced ferroptosis in glioblastoma cell lines U87MG and U251. NKAP expression was associated with the sensitivity of glioblastoma cells to the ferroptosis inducers erastin, iFSP1, and SAS. NKAP acted as a ferroptosis inhibitor by regulating the critical ferroptosis defense gene SLC7A11 through a posttranscriptional way. NKAP recruited SFPQ to perform TTS splicing events after binding to the “RGAC” motif of an m^6^A-containing site on SLC7A11 transcript.

## Results

### Cell death induced by NKAP knockdown could be blocked by ferrostatin-1 or alpha-tocopherol in glioblastoma cell lines U87MG and U251

**Fig. 1:**
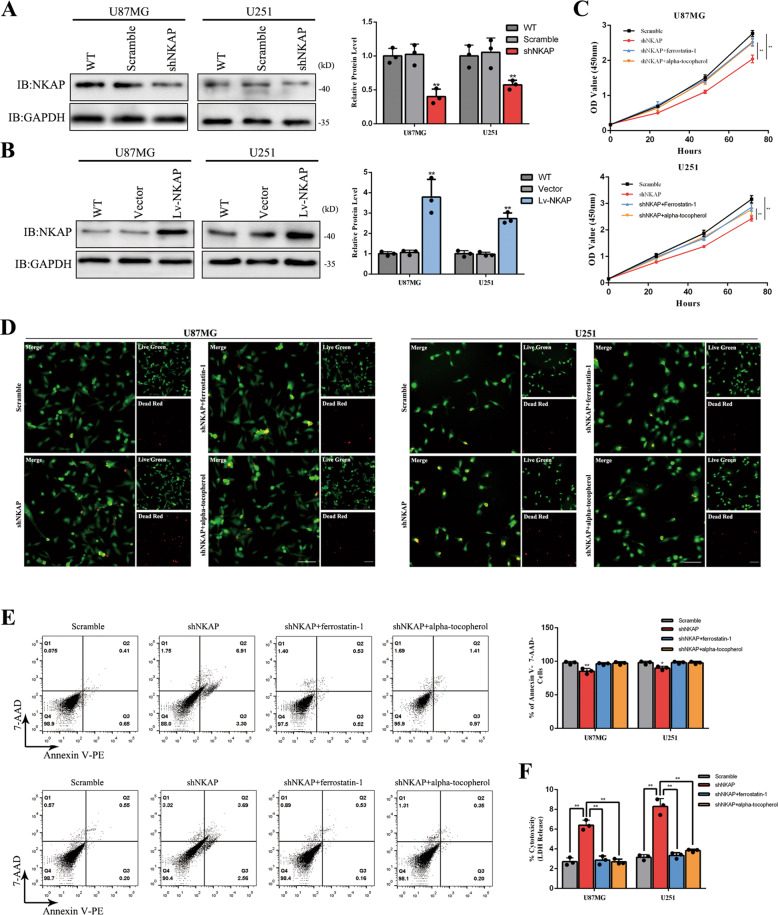
NKAP knockdown caused an increased percentage of cell death in glioblastoma cell lines. **A** The interference effect of shNKAP in U87MG and U251 cell lines. GAPDH was used as a loading control. **B** Protein overexpression level induced by Lv-NKAP in glioblastoma cell lines. GAPDH was used as a loading control. **C** The growth curves of the U87MG and U251 cells were examined using a CCK-8 assay. Cell growth was inhibited by NKAP knockdown and partially reversed by 0.5 μM ferrostatin-1 or 5 μM alpha-tocopherol for 24 h. **D** Live (green)/dead (red) staining revealed a higher cell death rate in the shNKAP group, which could be reversed by 0.5 μM ferrostatin-1 or 5 μM alpha-tocopherol for 24 h. Scale bar = 100 μm. **E** Cell death induced by NKAP knockdown in the absence or presence of 0.5 μM ferrostatin-1 or 5 μM alpha-tocopherol for 24 h detected by flow cytometry in U87MG and U251 cell lines. **F** LDH release induced by NKAP knockdown in the absence or presence of 0.5 μM ferrostatin-1 or 5 μM alpha-tocopherol for 24 h. All bars show the mean ± SD of three independent experiments. *P < 0.05, **P < 0.01.

We constructed a lentivirus-mediated RNAi targeting NKAP (shNKAP) and a lentiviral vector carrying the NKAP gene (Lv-NKAP) by transfecting to two human glioblastoma cell lines U87MG and U251, and then tested their interference effect and overexpression level by western blot (Fig. 1A, B). The CCK-8 assay was performed to evaluate whether NKAP could affect the cell proliferation rate. We found a significantly slower proliferation rate of the shNKAP group than scramble. Ferroptosis inhibitors ferrostatin-1 and alpha-tocopherol could partially reverse the effect (Fig. 1C). The change of cell viability was detected by live (green)/dead (red) staining. The shNKAP group exhibited more red fluorescence labeling than the scramble, which indicated a higher cell death rate (Fig. 1D). Ferrostatin-1 and alpha-tocopherol could almost completely reverse the effect. Apoptosis detection by flow cytometry revealed an average of 11% higher apoptotic rate in the shNKAP group than in the scramble group in U87MG. No obvious difference was observed between the Lv-NKAP group and vector. Moreover, U251 cells in the shNKAP group showed an average of 9% higher apoptotic rate than scramble. The cell death caused by NKAP knockdown could be blocked by ferrostatin-1 or alpha-tocopherol (Fig. 1E). The lactate dehydrogenase (LDH) release assay showed more LDH release in the cell culture medium in shNKAP group, which could also be reversed by ferrostatin-1 or alpha-tocopherol treatment (Fig. 1F).

### NKAP knockdown induced cell death by ferroptosis

**Fig. 2:**
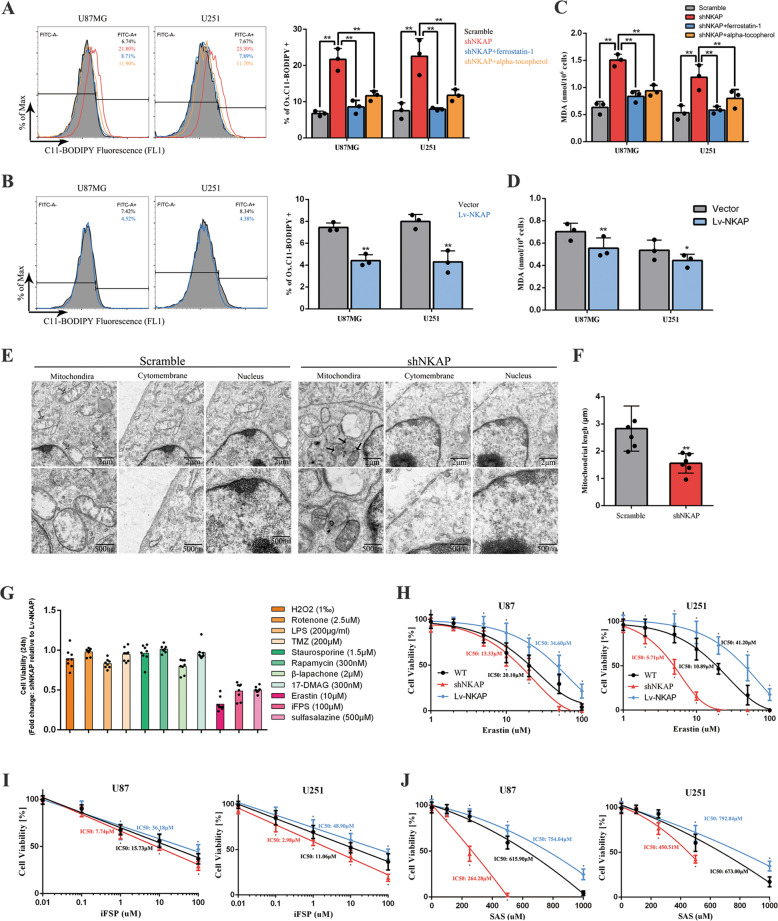
NKAP knockdown induced cell death through ferroptosis. **A** Lipid peroxidation level in U87MG and U251 cells was influenced by NKAP knockdown assessed by C11-BODIPY using flow cytometry immunolabelling in the absence or presence of 0.5 μM ferrostatin-1 or 5 μM alpha-tocopherol for 24 h. **B** Lipid peroxidation level influenced by NKAP overexpression. **C** MDA level influenced by NKAP knockdown in the absence or presence of 0.5 μM ferrostatin-1 or 5 μM alpha-tocopherol for 24 h. **D** MDA level influenced by NKAP overexpression. **E** Transmission electron microscopy revealed the characteristics of NKAP knockdown U87MG cells. The white arrow indicated normal mitochondria. Black arrow indicated deeply stained, shrunken mitochondria. **F** The longest diameter of the mitochondria in the scramble (*n* = 6) and shNKAP (*n* = 6) group. **G** Effect of NKAP on the U87MG cell sensitivity to erastin (10 μM), iFSP1 (100 μM), SAS (500 μM), rotenone (2.5 μM), 17-DMAG (300 nM), staurosporine (1.5 μM), TMZ (200 μM), β-lapachone (2 μM), H2O2 (1‰), LPS (200 μg/ml), and rapamycin (300 nM). All drug treatments were for 24 h. **H** Cell viability of U87MG and U251 glioma cells when grown in 1, 2, 5, 10, 20, 50, and 100 μM erastin for 24 h. **I** Cell viability when grown in 0.01, 0.1, 1, 10, and 100 μM iFSP1 for 24 h. **J** Cell viability when grown in 0, 100, 250, 500, and 1000 μM SAS for 24 h. All bars show mean ± SD of three independent experiments. *P < 0.05, **P < 0.01.

Given that there was a higher lipid peroxidation level in NKAP-deficient naive T cells [31], we hypothesized that NKAP knockdown induced cell death by ferroptosis. Oxidized C11-BODIPY was generally used as a ferroptosis marker. Flow cytometry immunolabelling with oxidized C11-BODIPY showed that NKAP knockdown significantly increased the lipid peroxidation level as compared to scramble, which could be blocked by ferrostatin-1 and alpha-tocopherol. (Fig. 2A). The statistical result of the C11-BODIPY-positive ratio was also shown on the right. In contrast, Lv-NKAP showed decreased lipid peroxidation level (Fig. 2B). The amount of malondialdehyde (MDA) increased, which indicated that NKAP knockdown cells had significantly higher lipid oxidation levels and more oxidative damage (Fig. 2C). In contrast, Lv-NKAP cells had a lower MDA level (Fig. 2D). Transmission electron microscopy showed typical ferroptosis ultrastructural changes, including deeply stained, shrunken mitochondria (left); ruptured cytomembrane (right), and normal nucleus without chromosome condensation (middle) in the shNKAP group in U87MG cells (Fig. 2E). We conducted quantification of mitochondria length in each group. Results showed shNKAP group cells had a significantly smaller mitochondrion than scramble group cells (Fig. 2F). We also conducted a JC-1 labeling assay to measure mitochondrial membrane potential which was one of the most important indicators of mitochondrial function [35]. Results showed NKAP knockdown induced lower mitochondrial membrane potential, which could be blocked by ferrostatin-1 or alpha-tocopherol (Supplementary Fig. S1). In summary, we found that glioblastoma cells with NKAP knockdown had a higher lipid peroxidation level and typical ferroptosis characteristics. Therefore, our hypothesis was verified: the cell death mode caused by NKAP knockdown was ferroptosis. Next, we used a small lethal drug bank that included inducers of different cell death modes: erastin, iFSP1, and SAS induced cell death by ferroptosis; rotenone, 17-DMAG, staurosporine, temozolomide (TMZ), and β-lapachone by apoptosis; H_2_O_2_ by necrosis; LPS by pyroptosis; and rapamycin by autophagic death. The results showed the cell viability ratio of Lv-NKAP to shNKAP at 24 h after the addition of the indicated drug dose. All four types of ferroptosis inducers caused significant differences in U87MG cell viability between the Lv-NKAP and shNKAP groups (Fig. 2G). Dose-dependent toxicity in the two glioblastoma cell lines was measured by the CCK-8 toxicity assay. Cell viability of U87MG and U251 glioma cell lines, when grown in the indicated concentration of erastin for 24 h, was significantly lower in the shNKAP group and higher in the Lv-NKAP group (Fig. 2H). An approximate curve was drawn to show the objective principle of the cytotoxic reaction. The same assays were performed to test the cytotoxic role of iFSP1, and SAS. The results showed the shNKAP group was more sensitive to all three ferroptosis inducers than the scramble group, while the Lv-NKAP group exhibited stronger resistance to these inducers than the vector group (Fig. 2I, J).

### NKAP targeted SLC7A11 during ferroptosis regulation

**Fig. 3:**
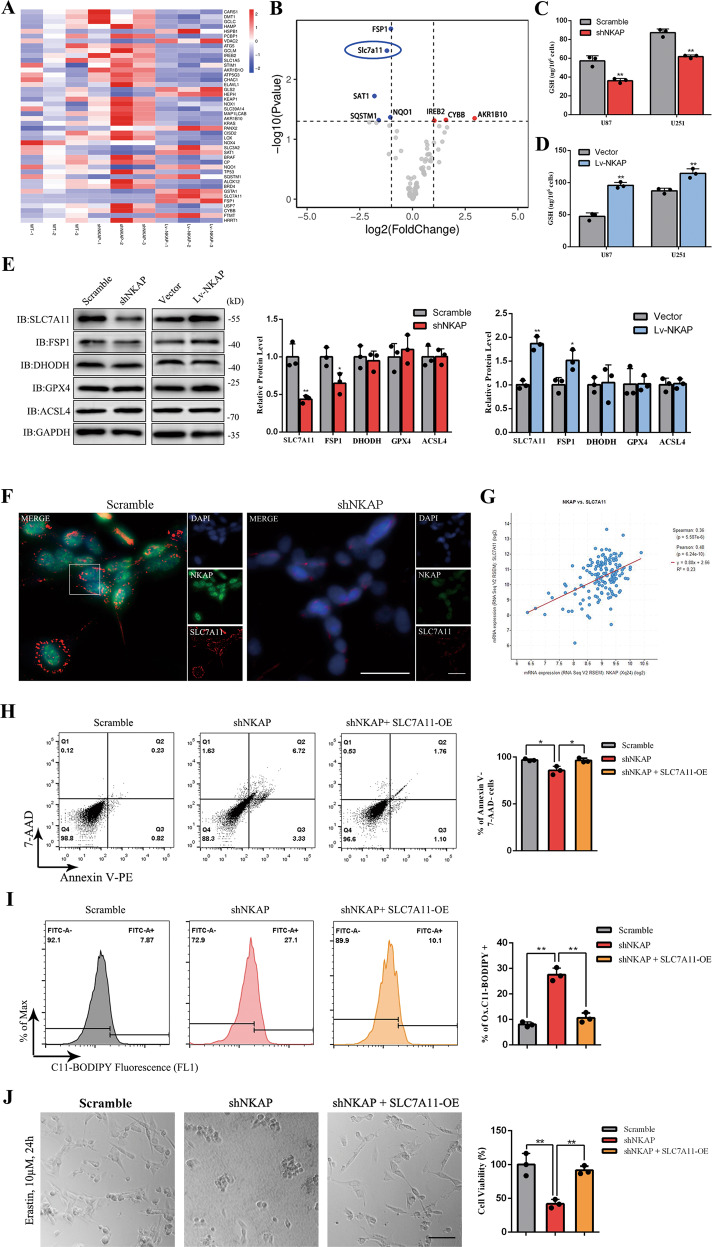
NKAP controlled ferroptosis by regulating the expression of SLC7A11. **A** Ferroptosis-related qPCR array of WT, shNKAP, and Lv-NKAP U87MG cells identified 44 differentially expressed genes. **B** The volcano map showed the difference in degree and *p* value, which identified SLC7A11 as the most relevant genes. **C** GSH level influenced by NKAP knockdown. **D** GSH level influenced by NKAP overexpression. **E** Protein levels of ferroptosis core genes measured by western blot assay. GAPDH was used as a loading control. **F** Immunofluorescence staining showed cell localization and visible expression changes between shNKAP and scramble groups. Scale bar = 50 μm. **G** Correlation between the expression of NKAP and SLC7A11 in clinical glioblastoma samples was assessed by transcriptome profiling in the TCGA clinical glioblastoma database. **H** Cell death induced by NKAP knockdown could be blocked by SLC7A11 overexpression detected by flow cytometry in U87MG. **I** High Lipid peroxidation level induced by NKAP knockdown could be reversed by SLC7A11 overexpression detected by flow cytometry in U87MG. **J** High drug sensitivity of NKAP knockdown U87MG cells to 10 μM erastin for 24 h could be reversed by SLC7A11 overexpression. Scale bar = 100 μm. All bars show mean ± SD of three independent experiments. *P < 0.05, **P < 0.01.

We used qPCR array to screen which ferroptosis-related molecules were downstream targets regulated by NKAP. Comparing the wild-type (WT), shNKAP, and Lv-NKAP groups in a U87MG cell line, all 44 significantly differentially expressed genes were listed in a heat map (Fig. 3A). Relative mRNA expression between samples with error bars was shown in Supplementary Fig. S2. Among all ferroptosis-related genes, SLC7A11 was identified as one of the most relevant genes based on the difference in fold change and *p* value, as shown in the volcano map (Fig. 3B). SLC7A11 acted as a cystine/glutamate antiporter to synthesize GSH and several articles reported that the downregulation of SLC7A11 could induce ferroptosis [36, 37]. We conducted an SLC7A11 knockdown assay to examine SLC7A11 was the downstream target of NKAP that mediated the effects observed (Supplementary Fig. S3). The shNKAP group showed a lower level of GSH than a scramble, while the Lv-NKAP group showed a higher level of GSH than vector (Fig. 3C, D). Protein expression of the ferroptosis core genes GPX4, FSP1, DHODH, and ACSL4 was also measured by western blot assay (Fig. 3E). Immunofluorescence staining was used to visualize the expression changes and the localization of SLC7A11 accompanying NKAP knockdown and in U87MG cells (Fig. 3F). Transcriptome profiling was performed using the TCGA clinical glioblastoma database (Public data, Project ID: TCGA-GBM, 166 glioblastoma cases) to investigate the correlation between NKAP and SLC7A11. The results showed a positive correlation between NKAP and SLC7A11 (Fig. 3G). In the double transfection test assay, the shNKAP + SLC7A11-overexpression plasmid (shNKAP + SLC7A11-OE) group showed a significantly lower death rate (Fig. 3H), less C11-BODIPY positive cells (Fig. 3I), and stronger resistance to the ferroptosis inducer erastin than the shNKAP group in U87MG cells (Fig. 3J). In conclusion, we found that NKAP functioned as a ferroptosis suppressor by regulating SLC7A11 expression.

### NKAP knockdown suppressed glioblastoma growth and increased the sensitivity of cells to ferroptosis inducer in vivo

**Fig. 4:**
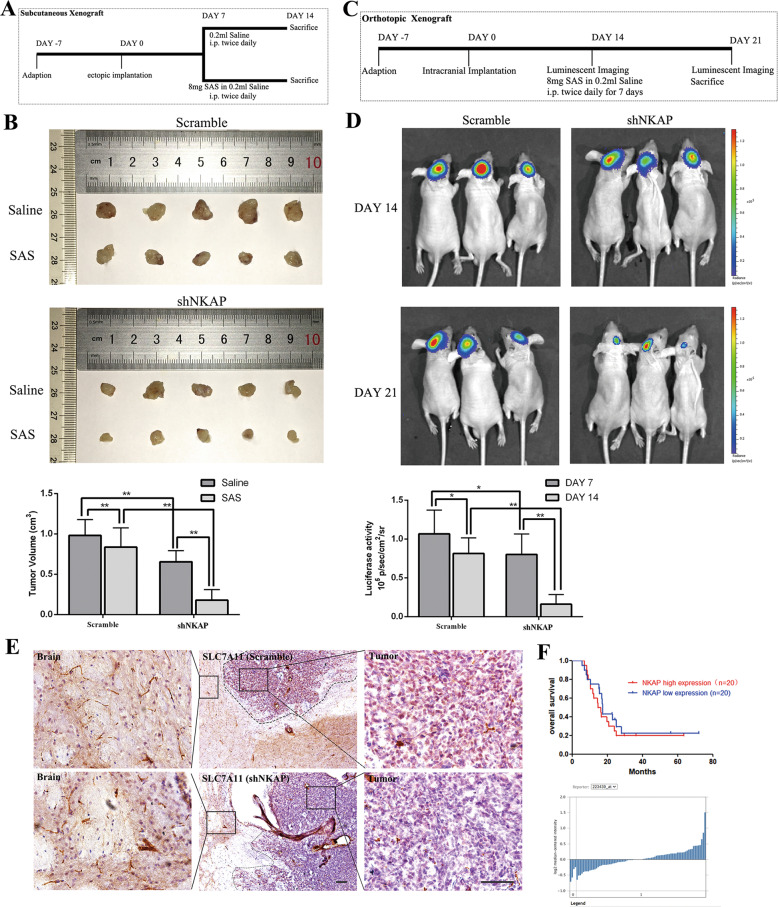
NKAP suppressed glioblastoma growth and reduced sensitivity to ferroptosis inducer in vivo. **A** Scheme showing the design of the subcutaneous xenograft model. **B** Representative subcutaneous xenograft tumor 14 days after inoculation showed reduced tumor volume and better efficacy of SAS in the shNKAP group. The bar graph indicates mean ± SD values of six independent animals. **C** Schematic showing the design of a subcutaneous xenograft model in an orthotopic intracranial mouse model. **D** Representative bioluminescence images of the intracranial tumor showed decreased luciferase activity and better efficacy of SAS in the shNKAP group. The bar graph indicates mean ± SD values of six independent animals. **E** Immunohistochemical labeling showed the downregulated expression of SLC7A11 in the shNKAP group. Scale bar = 100 µm. **F** Curves showed the better curative effect of standard therapy of glioblastoma patients in the NKAP high expression group. The graph below showed the relative NKAP expression level in 40 samples. *P < 0.05, **P < 0.01.

Because of the nephrotoxicity of erastin and iFSP1, we used SAS as the ferroptosis inducer in vivo. We designed a series of experiments to investigate the role of NKAP in vivo. Scramble and shNKAP U87MG cells transfected with luciferase were subcutaneously implanted into immunocompromised mice to develop a subcutaneous xenograft model. The animal experimental process for each group was shown (Fig. 4A). We observed a decreased tumor volume in the tumor-bearing mice when NKAP was inhibited. Treatment with SAS (8 mg, intraperitoneal dose, twice daily for 7 days) decreased the tumor volume by nearly 20% in the scramble group and by ~70% in the shNKAP group (Fig. 4B). Moreover, as shown in Fig. 4C, in an orthotopic intracranial mouse model, the tumor size was slightly reduced in the U87MG-Luc/shNKAP group on day 14. After 7 days of treatment with SAS, the luciferase activity of the U87MG-Luc/shNKAP group decreased more obviously than the U87MG-Luc/scramble group (Fig. 4D). Immunohistochemical staining of SLC7A11 and FSP1 revealed a much lower expression of SLC7A11 in the shNKAP group than in the peritumoral brain tissue (Fig. 4E). The standard therapy for newly diagnosed glioblastoma was radiotherapy with concurrent temozolomide (TMZ) administration following maximal safe resection of the tumor. Many reports have shown that radiotherapy induces tumor cell death by ferroptosis [38, 39]. Therefore, we investigated the relationship between the NKAP expression level and the curative effect of standard therapy by retrieving public data from the ONCOMINE Dataset (dataset number: GSE7696), in which we selected 20 samples with the highest NKAP expression level and 20 samples with the lowest expression. The results showed a higher survival rate in NKAP low expression group after standard therapy, but no significant difference was found, which might be due to the small sample size and a small difference in the expression level of NKAP in human glioblastoma samples (Fig. 4F).

### NKAP acted as an m^6^A reader and promoted SLC7A11 expression

**Fig. 5:**
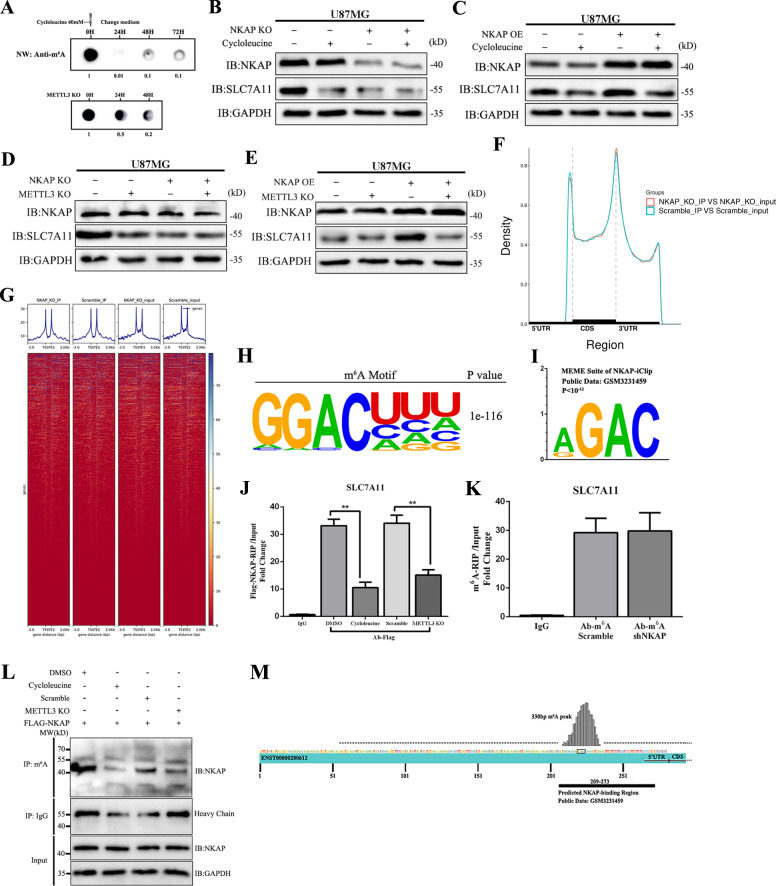
Binding to m^6^A was necessary for NKAP to promote SLC7A11 expression. **A** m^6^A northwestern blot assay was performed using an equal amount of RNA (1 μg) isolated from each group. Briefly, the cells were treated with 40 μM cycloleucine for 24 h until the change of culture media. **B** Western blot assay showed SLC7A11 protein expression regulated by NKAP knockdown and 40 μM cycloleucine for 24 h. **C** SLC7A11 protein expression regulated by NKAP overexpression and 40 μM cycloleucine for 24 h. **D** SLC7A11 protein expression is regulated by NKAP knockdown and METTL3 knockdown. **E** SLC7A11 protein expression regulated by NKAP overexpression and METTL3 knockdown. **F** Similar m^6^A peak density in the 5ʹ-UTR, CDS, and 3ʹ-UTR regions in the scramble and shNKAP groups. **G** Enrichment of reads near the transcription start site (TSS) and transcription end site (TES). **H** HOMER identified the m^6^A consensus motif in the U87MG cells as “RGACH”. **I** MEME SUIT analyzed NKAP footprints as the “RGAC” motif based on the NKAP-iCLIP public data. **J** FLAG-NKAP-RIP revealed that enrichment of SLC7A11 mRNA significantly decreased in the cycloleucine-treated and METTL3 knockdown groups. **K** m^6^A-RIP showed no significant difference in SLC7A11 mRNA enrichment between the scramble and shNKAP groups. **L** Co-IP analysis of the interaction between m^6^A and Flag-NKAP in U87MG cells. **M** m^6^A-seq showed that the m^6^A peak in the SLC7A11 transcript ENST00000613322 had an overlap with the predicted NKAP binding region. GAPDH was used as an internal control in western blot. All bars show mean ± SD of three independent experiments. *P < 0.05, **P < 0.01.

Next, we investigated the mechanism under NKAP regulating SLC7A11 in U87MG cells. We used a global m^6^A inhibitor cycloleucine and a knockdown assay of m^6^A writer methyltransferase-like 3 (METTL3) to investigate whether NKAP modulated downstream expression by m^6^A modification. Northwestern blot using an m^6^A-specific antibody showed a significantly decreased global m^6^A level following treatment with cycloleucine or METTL3 knockdown at the indicated time points (Fig. 5A). Western blot showed a similar decline in the levels of SLC7A11 protein in the NKAP knockdown group with cycloleucine treatment group (40 μM, 24 h) (Fig. 5B). SLC7A11 protein level decreased to a low level when the cells were treated with cycloleucine (Fig. 5C); NKAP influenced the protein levels of SLC7A11 depending on METTL3 expression, and METTL3 knockdown blocked the function of NKAP (Fig. 5D, E). Based on the results, we found that m^6^A modification was necessary for NKAP’s regulation effect on SLC7A11. To further investigate the precise role of m^6^A in the regulation process of NKAP, we conducted m^6^A-sequence in the scramble and shNKAP U87MG cells. The results showed a similar m^6^A peak density in the 5ʹ-UTR, CDS, and 3ʹ-UTR regions (Fig. 5F), which suggested that NKAP could not influence the formation and elimination of m^6^A modification. We combined all the peak reads together to show the enrichment of reads were near the transcription start site (TSS) and transcription end site (TES) (Fig. 5G). The m^6^A motif is a nucleic acid sequence pattern with multiple biological functions and can be identified by some RNA-binding proteins. The m^6^A consensus motif in the U87MG cells was identified as “RGACH” by HOMER software based on our m^6^A-sequence (Fig. 5H). High enrichment of the “RGAC” motif of NKAP footprints was analyzed by the MEME SUIT web server [40] based on the NKAP-iCLIP public data (GSM3231459) (Fig. 5I). The similar motifs of m^6^A and NKAP footprints suggested the molecular binding of NKAP to the m^6^A-containing site of the SLC7A11 transcript. To verify the direct binding of NKAP to SLC7A11 mRNA transcript, we used the FLAG-NKAP plasmid and Ab-FLAG to precipitate mRNA, which was quantitatively detected by qPCR. The degree of enrichment of SLC7A11 mRNA was measured by the ratio of RIP to input. The results showed a significantly decreased enrichment in the cycloleucine and METTL3 knockdown groups as compared to that in control, which suggested that m^6^A modification was necessary for binding (Fig. 5J). However, m^6^A-RIP showed no significant difference in the enrichment of both SLC7A11 mRNA between the shNKAP group and the scramble group (Fig. 5K). In summary, m^6^A can affect the function of NKAP, but NKAP cannot affect the formation or elimination of m^6^A. To verify that NKAP was an m^6^A reader, we used an m^6^A antibody to precipitate the NKAP protein. The results showed that NKAP directly bound to m^6^A-containing mRNA and interferes with m^6^A (Fig. 5L). Finally, we blasted the NKAP-iCLIP data with the SLC7A11 transcript (ENST00000280612) sequence and found the predicted binding region of NKAP that showed good overlap with a 330-bp m^6^A peak on the 5ʹ-UTR of SLC7A11 transcript detected by m^6^A-sequence (Fig. 5M). These findings revealed that NKAP was directly bound to the m^6^A-containing site of SLC7A11 transcript in advance and then increased SLC7A11 protein level.

### NKAP regulated TTS alternative splicing event and retention of the last exon

**Fig. 6:**
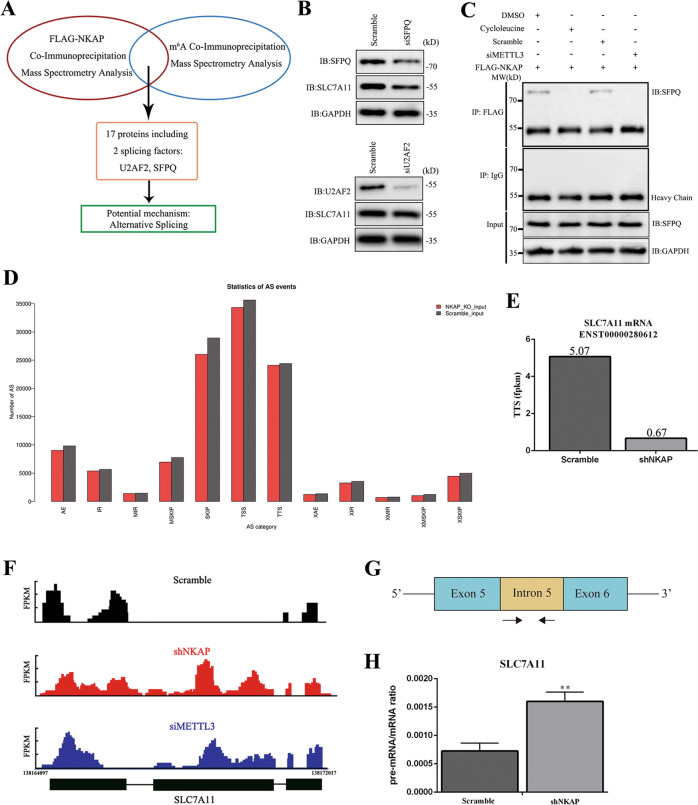
NKAP recruited SFPQ to regulate the TTS splicing event and mRNA maturation. **A** Schematic of Co-IP experiment and proteomic screening by MS to identify the potential mechanism. **B** SFPQ knockdown decreased protein levels of SLC7A11. U2AF2 knockdown had no effect on SLC7A11 expression. **C** Co-IP analysis of the interaction between Flag-NKAP and SFPQ, which could be blocked by treatment with either cycloleucine or METTL3 RNAi. **D** Chart showed ASprofile analysis and statistics of alternative splicing events for all transcripts based on high-throughput RNA sequencing. **E** Decreased occurrence of alternative transcription termination site (TTS) on the SLC7A11 transcript. **F** Sashimi plots showing retained exon on the SLC7A11 transcript in both shNKAP and siMETTL3 groups. **G** Schematic representation of strategy to identify intron (unspliced, pre-mRNA). **H** qPCR showed that the pre-mRNA/mRNA ratio of SLC7A11 increased significantly in the shNKAP group. GAPDH was used as an internal control in western blot. All bars show mean ± SD of three independent experiments. *P < 0.05, **P < 0.01.

We conducted proteomic screening by mass spectrum (MS) using the products of FLAG-NKAP Co-IP and m^6^A Co-IP to identify the underlying mechanism. Seventeen proteins were found to potentially interact with NKAP and m^6^A-containing mRNA (Supplementary Table 1). Two splicing factors, namely U2 small nuclear RNA auxiliary factor 2 (U2AF2) and SFPQ, were screened out (Fig. 6A). We found that SFPQ knockdown could decrease SLC7A11 protein level, but U2AF2 knockdown could not; this finding suggested that the function of NKAP was related to SFPQ but not U2AF2 (Fig. 6B). Given that NKAP was reported to widely participate in posttranscriptional modulation by alternative splicing [20, 21] and that the m^6^A mark was implicated in pre-mRNA processing [24, 41–43], we hypothesized that NKAP recruited the splicing factor SFPQ to recognize alternative splice site after binding to m^6^A site. SFPQ was a well-known splicing factor and participated in an oncogenic transcriptomic state [44, 45]. We performed Co-IP to investigate the interaction between Flag-NKAP and SFPQ. We found that the interaction could be blocked by treatment with either cycloleucine or METTL3 RNAi, which implied that NKAP recruited SFPQ after binding to m^6^A (Fig. 6C). Alternative splicing events mainly included exon skipping (SKIP) and cassette exons (MSKIP), retention of single (IR) and multiple (MIR) introns, alternative exon ends (AE), alternative transcription start site (TSS), and alternative transcription termination site (TTS) [46, 47]. We used ASprofile to perform qualitative and statistical analysis of alternative splicing events for all transcripts predicted by Stringtie based on the RNA-sequence (Fig. 6D). The analysis of splicing event changes of the SLC7A11 transcript showed a significant decrease in the occurrence of TTS as measured by FPKM (Fig. 6E). Next, we used MISO [48] to quantify the relative expression of alternatively spliced exons in the scramble, NKAP knockdown, and METTL3 knockdown cells. We found that NKAP and METTL3 regulated the common alternative splicing event TTS, and the same exon was retained in the SLC7A11 transcript (Fig. 6F). We attempted to design the specific primer of SLC7A11 pre-mRNAs. We designed a pair of primers reflecting SLC7A11 pre-mRNA level in U87MG. The primer of SLC7A11 pre-mRNA amplified intron 5 sequence of NM_011990 transcript (Fig. 6G). The pre-mRNA/mRNA ratio of shNKAP cells increased apparently, which suggested the failure of alternative splicing (Fig. 6H).

## Discussion

New technical advances in molecular biology have revealed widespread and different types of modifications of mRNA [49]. m^6^A was a highly pervasive modification of mRNA that affects RNA splicing, translation, and stability [50, 51]. The effects of m^6^A were determined by m^6^A writers, erasers, and readers [52]. m^6^A modification has been reported to widely participate in the progression of various cancers, including glioblastoma [53]. METTL3-mediated m^6^A modification played a crucial role in glioma stem-like cell maintenance and radioresistance [54]. m^6^A altered mRNA structure to enhance the binding of the m^6^A reader HNRNPC [41], which could affect the translation status and lifetime of mRNA [42]. m^6^A modification could be blocked specifically by cycloleucine [55–57]. We explored the effect of cycloleucine on ferroptosis. The results showed cycloleucine could not cause cell death or increase oxidized C11-BODIPY level (Supplementary Fig. S4A, B). In our model, NKAP regulated SLC7A11 splicing through m^6^A, and NKAP could not bind to SLC7A11 transcript without m6A. For this phenomenon, we speculate that m^6^A modification may affect the level of ferroptosis through different pathways (including positive and negative pathways)—that is, there may be other promotion pathways to compensate for the inhibition of ferroptosis by NKAP through m^6^A modification. At present, the research on m^6^A and ferroptosis is relatively limited, which has reported that m^6^A modification can upregulate the level of ferroptosis in hepatocellular carcinoma and lung adenocarcinoma. For example, Shen et al reported that m6A regulated ferroptosis through autophagy signaling pathway in hepatic stellate cells [58]. Ma et al. reported that m^6^A reader YTHDC2 induced ferroptosis by targeting SLC3A2 in lung adenocarcinoma [59]. However, the regulatory mechanism of m^6^A on ferroptosis in glioblastoma has not been reported, more interestingly, we observed that the m^6^A “writer” (METTL3) and “eraser” (ALKBH5) were both positively correlated with SLC7A11 in glioblastoma by analyzing TCGA data (Supplementary Fig. S4C), suggesting that m^6^A modification may regulate ferroptosis through both positive and negative pathways in GBM. In summary, the effect of m^6^A on ferroptosis is a complex process that requires more research.

In the present study, we first identified the subtle relationship between NKAP, ferroptosis, and m^6^A modification in glioblastoma cells. NKAP acted as a ferroptosis inhibitor by regulating the critical ferroptosis defense gene SLC7A11 in an m^6^A-dependent manner. NKAP is directly bound to the m^6^A-containing site, which was a precondition for NKAP to regulate SLC7A11. RNA-seq and MS showed that NKAP recruited SFPQ and controlled TTS splicing events in the presence of m^6^A. NKAP has not yet been reported to be involved in SLC7A11 mRNA splicing and maturation in glioblastoma cells, and there is no mention of its association with SFPQ to regulate the TTS event. Apart from TTS, NKAP controls many other splicing events in the entire genome, but no difference was found for the SLC7A11 transcript.

**Fig. 7:**
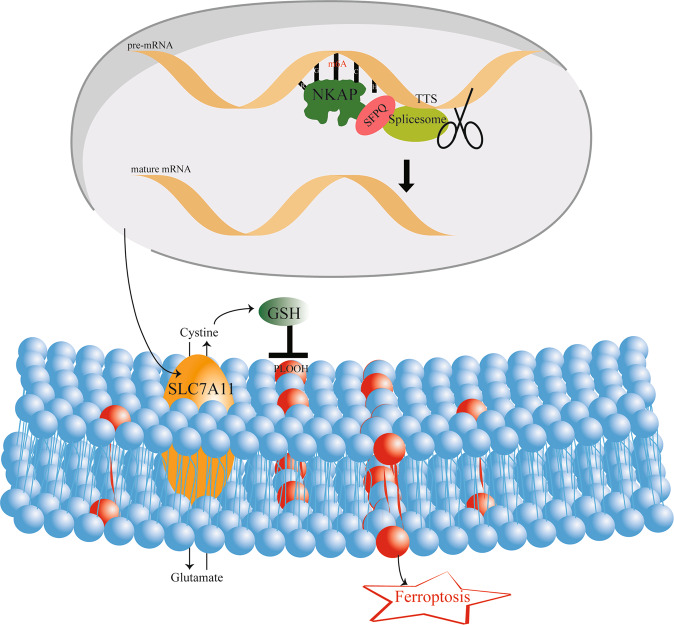
Pattern diagram of NKAP modulation of ferroptosis. NKAP functions as an m^6^A reader and recruits a splicing complex to promote the TTS event on the SLC7A11 transcript to produce mature mRNA. SLC7A11 anchored in the cytomembrane mediates the entry of cystine to generate GSH, which can neutralize PLOOH and ROS and eventually prevents ferroptosis.

Previous studies have reported NKAP knockout increased lipid peroxidation in naive T cells and induced cell death in colon cancer cells. However, there is no literature reporting the relationship between NKAP and ferroptosis in glioblastoma cells and the mechanism of NKAP modulating ferroptosis. We found a new regulatory mechanism of m^6^A in ferroptosis. The link between them was NKAP. We identified NKAP as a new ferroptosis suppressor, and the knockdown of NKAP directly led to ferroptosis in glioblastoma cells. NKAP protected glioblastoma cells from ferroptosis by positively regulating SLC7A11 expression. Mechanically, NKAP acted as an m^6^A reader; it first bound to the “RGAC” motif of m^6^A-containing site, then recruited SFPQ to promote TTS alternative splicing event, and eventually promoted mRNA maturation (Fig. 7). NKAP knockdown combined with ferroptosis induction therapy may be an effective treatment for glioblastoma in the future.

## Materials and methods

### Cell culture and transfection

Two human cell lines (U87MG, U251) were obtained from the Cell Bank of Type Culture Collection of the Chinese Academy of Sciences (Shanghai, China) and used within 6–8 months. All cells had tested for mycoplasma contamination and were cultured in high glucose Dulbecco’s modified Eagle’s medium (DMEM, Gibco, USA) with 10% fetal bovine serum (FBS, Biological, Industries) and 1% penicillin-streptomycin (10378016, Invitrogen, USA) at 37 °C with 5% CO_2_. Plasmids and siRNA were transfected into cells with Lipofectamine 3000 (L3000-008, Invitrogen, USA) according to the manufacturer’s instructions. lentivirus particles were directly added into target cells with 4 μg/ml polybrene (GM040901, Genomeditech, Shanghai, China). After two rounds of infection, cells were selected for at least 7 days by adding 10 μg/ml blasticidin (B9300, Solarbio Life Sciences, Beijing, China) or 1 μg/ml puromycin (P8230, Solarbio Life Sciences, Beijing, China) into the growth medium.

### Lentivirus, plasmid, and short interfering RNAs

NKAP-targeting short hairpin RNAs (ACAGUGACUCUGAUUCUGAAACAGA for humans and GGAAAUCUAGUCAUUCAAAAGACAG for mouse) and a nonspecific control (scramble, TTCTCCGAACGTGTCACGT) were constructed using a pGMLV-hU6-MCS-CMV-Blasticidin-WPRE lentiviral vector. The pGMLV-CMV-MCS-3xFLAG-PGK-Puro vector was used to construct an NKAP-overexpressing lentivirus. The pGMLV-CMV-MCS-EF-ZsGreen1-T2A-Puro vector was used to construct a SLC7A11 overexpressing plasmid. A short interfering RNA (siRNA) was used to target METTL3 (GGACCAAGGAAGAGUGCAU), U2AF2 (GAAACACCUCAAGUGGAGACA), SFPQ (GGAAAGACAAGCAUUAGAAA), and SLC7A11 (CCAUUAUCAUUGGCACCAUTT). Lentivirus, plasmid, and siRNAs were purchased from Genomeditech, Shanghai, China.

### Western blot and northwestern blot

Total proteins were extracted using RIPA Lysis Buffer (P0013C, Beyotime Biotechnology, Shanghai, China) containing protease/phosphates inhibitors (P1050, Beyotime Biotechnology, Shanghai, China). An equal amount of protein samples from each group and a standard molecular weight marker were loaded on a 10% SDS- PAGE gel, followed by transferred to polyvinylidene difluoride (PVDF) membranes and blocked with 5% skimmed milk for 2 h. Membranes were probed with primary antibodies subsequent with horseradish peroxidase-conjugated secondary antibodies followed by development using a super-sensitive electrochemiluminescence (ECL) reagent (WBULS0100, Millipore, USA) and an enhanced chemiluminescence detection system (Amersham Imager 680). Blots were sometimes stripped using stripping buffer (P0025N, Beyotime Biotechnology, Shanghai, China) and probed with other primary antibodies. The densitometric quantification was analysed with a GAPDH control using ImageJ software (Bio-Rad, Hercules, CA, USA). Northwestern blot assay was taken followed by springer protocols. The primary antibodies were rabbit polyclonal anti-NKAP (ab229096, Abcam, USA), rabbit polyclonal anti-SLC7A11/xCT (26864-1-AP, Proteintech, Wuhan, China), goat polyclonal anti-SLC7A11/xCT (ab60171, Abcam, USA), rabbit polyclonal anti-AIFM2/FSP1 (20886-1-AP, Proteintech, Wuhan, China), rabbit polyclonal anti-ACSL4 (A16848, ABclonal, China), rabbit polyclonal anti-GPX4 (14432-1-AP, Proteintech, Wuhan, China), rabbit polyclonal anti-DHODH (14877-1-AP, Proteintech, Wuhan, China), rabbit polyclonal anti-GAPDH (10494-1-AP, Proteintech, Wuhan, China), rabbit monoclonal anti-m^6^A (D6W5B, Cell Signaling, USA), rabbit polyclonal anti-SFPQ (15585-1-AP, Proteintech, Wuhan, China), rabbit polyclonal anti-U2AF2 (15624-1-AP, Proteintech, Wuhan, China). The second antibody was HRP-conjugated affinipure goat anti-rabbit (SA00001-2, Proteintech, Wuhan, China).

### Cell proliferation assay

Cell proliferation was determined using a Cell Counting Kit-8 (CCK-8) kit (HY-K0301, MCE, USA). Scramble and shNKAP cells of U87MG and U251 cell lines were seeded into 96-well plates for 0, 24, 48, and 72 h at a density of 3000 cells per well after treatment with 0.5 μM ferrostatin-1 (HY-100579, MCE, USA) or 5 μM alpha-tocopherol (HY-16686, MCE, USA) for 24 h or not. Then, 10 μL CCK-8 solution was added to each well and incubated with the cells for 2 h. Absorbance was detected at 450 nm using a microplate reader (Bio-Rad, Hercules, CA, USA).

### Cell viability

U87MG cells of the WT group, shNKAP group, and Lv-NKAP group were seeded on 96-well plates until they reached ~50–60% confluency the next day. Each group of cells were treated with compounds including 10 μM erastin (HY-15763, MCE, USA), 100 μM iFSP1 (HY-136057, MCE, USA), 500 μM SAS (HY-14655, MCE, USA), 2.5 μM rotenone (HY-B1756, MCE, USA), 300 nM 17-DMAG (HY-10389, MCE, USA), 1.5 μM staurosporine (HY-15141, MCE, USA), 200 μM TMZ (HY-17364, MCE, USA), 2 μM β-lapachone (HY-13555, MCE, USA), 1‰ H2O2, 200 μg/ml LPS (HY-D1056, MCE, USA), and 300 nM rapamycin (HY-10219, MCE, USA) for 24 h. Cell viability was assessed 24 h later by CCK-8. Results were shown as the ratio: (450 nm absorbance in shNKAP/450 nm absorbance in WT)/(450 nm absorbance in Lv-NKAP/450 nm absorbance in WT). Next, cells were treated with increasing concentrations of selected drugs (erastin, iFSP1, SAS) to draw an approximate dose-dependent toxicity curve.

### Live/dead assay

We used the LIVE/DEAD Cell Imaging Kit (R37601, Invitrogen, USA) to visualize the cell’s live/dead state. The red component in the kit was cell impermeable, so it can only enter the cells with a damaged cell membrane. When it binds to DNA in dying and dead cells, it produces bright red fluorescence. Transfer component A into component B to make a 2×working solution, which was added in different groups for 15 min at 20–25 °C. Treatment with 0.5 μM ferrostatin-1 or 5 μM alpha-tocopherol for 24 h.

### Evaluation of malondialdehyde (MDA), lactate dehydrogenase (LDH), and glutathione (GSH) level

In cells, free radicals reacted with lipid to produce peroxidation, and the final product of oxidation was malondialdehyde. The MDA concentration was assessed using the Micro Malondialdehyde (MDA) Assay Kit (BC0025, Solarbio, Beijing, China) according to the manufacturer’s protocol. LDH is a cytoplasmic enzyme that is retained by viable cells with an intact plasma membrane and released from cells with damaged membranes. LDH release was measured as described in the protocol: Analysis of Cell Viability by the Lactate Dehydrogenase Assay. Maximum LDH release levels were measured by treating the cultures with 10x lysis solution to measure complete lysine levels in the cells. Absorbance was measured at a 490 nm microplate reader. Determine the percent of cell death (% cytotoxicity) using the following equation: % Cytotoxicity = Experimental LDH release (OD490) Maximum LDH release (OD490). Treatment with 0.5 μM ferrostatin-1 or 5 μM alpha-tocopherol for 24 h. Micro Reduced Glutathione (GSH) Assay Kit (BC1175, Solarbio, Beijing, China) was used to measure glutathione levels in 50,000 cells per replicate following the manufacturer’s instructions.

### RNA extraction and quantitative real-time PCR array

Total RNA was extracted from cultured cells using a Total RNA extraction kit (RC112-01, Vazyme Biotech, Nanjing, China). The concentration and purity of RNA were measured by the absorbance at 260 nm and the ratio of 260/280 nm in NanoDrop ND-1000 (NanoDrop, Wilmington, DE, USA). Total RNA from each sample was reversely transcribed using an all-in-one cDNA synthesis superMix (R333-01, Vazyme Biotech, Nanjing, China). SYBR Green PCR kit (R311-02, Vazyme Biotech, Nanjing, China) was used for real-time PCR. The primers of related genes are listed in Supplementary Table 2. Quantitative PCR arrays are designed to analyse a panel of ferroptosis-related genes in human glioblastoma cell line U87MG following the instructions of the manufacturer (Wcgene Biotechnology Corporation, China). Genes undetectable for three times were excluded. The Ct values of each gene were corrected by Ct reading of corresponding GAPDH. The reaction was performed using Light Cycler 480 (Roche).

### Immunofluorescence (IF) and immunohistochemistry (IHC)

U87MG cells were grown on cover slides, fixed, blocked with 3% BSA, and permeabilized with PBS containing 0.1% w/v Triton X-100. The mouse brain of the orthotopic intracranial mouse model was cut in the thickness of 30 µm by a frozen section and blocked with 3% BSA. For protein detection, the primary antibodies used have been already described in the western blot section. For IF, Secondary antibodies were goat IgG anti-rabbit or mouse conjugated to a fluorochrome. Nuclei were stained by 4,6-diamidino2-phenylindole (DAPI). Image acquisition was performed on ImageXpress Micro Confocal Devices. IHC staining was performed by using Immunofluorescence Two-Step Test Kit (PV-9000, ZSGB-BIO, Beijing China). Image acquisition was performed on an OLYMPUS BX63 fluorescence microscope.

### Transmission electron microscopy

Collect cells precipitation after centrifuge. The IEM fixative was added to the tube and let the precipitation resuspended in the fixative, and then fixed at 4 °C for preservation. The 2% low melting point agarose solution was prepared by heating and dissolving in advance. After being cooled to about 40 °C, the agarose solution was added to the EP tube. Before agarose solidification, the precipitation was suspended with forceps and wrapped in the agarose. Then the sample was put into the pure resin and the capsule cap was covered. After vacuum extraction for 0.5–1 h, the capsule cap was fastened for embedding. The capsules with resin and samples were moved into a low-temperature UV polymerizer to polymerize for more than 48 h at −20 °C. And then the resin blocks were taken out from the capsules for standby application at room temperature. The resin blocks were cut to 70–80 nm thin on the ultra-microtome, and the tissues were fished out onto the 150 meshes nickel grids with formvar film. The nickel grids with tissues were stored at 4 °C for standby application. The nickel grids are observed under TEM and take images. The longest diameter of the mitochondria was measured by ImageJ software.

### Flow cytometry

Cell samples were detached with trypsin and washed 1–2 times with flow cytometry staining buffer. Resuspend cells in an appropriate volume of flow cytometry staining buffer about 500 μl. The protocol described here of apoptotic staining had been run on Flow Cytometer BD FACSCanto™ II (BD Bioscience, Heidelberg, Germany). For every 100 μl of cell suspensions, add 5 μl of PI working solution (final concentration of PI is 5 μg/ml) or 7-AAD staining solution (final concentration 1 μg/ml). Incubate cells for 15 min in a light-protected box on ice before analyzing cells on the flow cytometer. Lipid peroxidation was assessed using BODIPY-C11 as the probe (D3861, Thermo, USA). This probe readily incorporates into membranes where it responds to attack by peroxyl radicals by undergoing a spectral emission shift from red to green. This change can be readily monitored and quantified by flow cytometry. Bodipy-C11 (working concentration: 5 uM) was added to the culture medium, incubated for 30 min at 37 °C. Then remove the media and wash cells three times in PBS. The FL-1 (530 nm bandpass filter) was used to measure green fluorescence luminosity by Flow Cytometry. Treatment with 0.5 μM ferrostatin-1 or 5 μM alpha-tocopherol for 24 h. At least 10,000 events were analyzed per sample. Each sample was analyzed in triplicate. Quantification and analysis of the results were done with FlowJo_V10 (Tree Star, Ashland, USA).

### Subcutaneous xenograft model and orthotopic intracranial mouse model

All experimental animal procedures were conducted strictly by the Guide for the Care and Use of Laboratory Animals and approved by the Animal Care and Use Committee of the Shandong provincial hospital affiliated to Shandong University. The sample size of the animal experiment was based on the preliminary experiments and similar well-designed experiments, and no statistical method was used. The male BALB/c nude mice were randomized divide into two groups, each group including six 4 weeks old nude mice. Investigators were blinded to the treatment groups during data collection and subsequent data analysis. In the subcutaneous xenograft model, 5 × 10^5^ cells were subcutaneously injected in the right flanks of nude mice. In the orthotopic intracranial mouse model, each mouse was intracranially injected with 1 × 10^5^ luciferase transfected U87MG cells in 10 μL PBS solution. Tumor growth was monitored by a Xenogen IVIS Spectrum system (PerkinElmer).

### RIP

RIP was performed using the EZ-Magna RIP^TM^ RNA-Binding Protein Immunoprecipitation Kit (Cat.# 17-701, Millipore, America). The experimental procedure was as follows: 4 × 10^7^ cells were harvested and resuspended in 200 µl RIP lysis buffer (100 µl RIP lysis buffer containing 0.5 µl protease inhibitor cocktail and 0.25 µl RNAse inhibitor). The magnetic beads were coated with an antibody (Ab-FLAG: D9D9W, Cell Signaling, America; Ab-m^6^A: D6W5B, Cell Signaling, America; IgG for control) to prepare them for immunoprecipitation. The RIP lysate was centrifuged at 14,000 rpm for 10 min at 4 °C. Next, 100 µl of the supernatant per immunoprecipitation was removed and added to tubes containing the bead-antibody complex in a RIP immunoprecipitation buffer (860 µl RIP wash buffer, 35 µl 0.5 M EDTA, 5 µl RNAse inhibitor). All the tubes were incubated under rotation at 4 °C for overnight. The immunoprecipitation tubes were centrifuged and then placed on a magnetic separator, and the supernatant was then discarded. The beads were washed six times. Each immunoprecipitate was then resuspended in 150 µl proteinase K buffer (107 µl RIP wash buffer, 15 µl 10% SDS, 18 µl proteinase K), and all tubes were incubated at 55 °C for 30 min under shaking to digest the protein. The tubes were then centrifuged and placed on a magnetic separator to collect the supernatant. The RNA was extracted by the phenol-chloroform method and precipitated with ethanol.

### Co-IP

For Co-IP assay, cells were collected 48 h after transfection and lysed in RIPA Lysis Buffer supplemented with protease/phosphates inhibitors (P1050, Beyotime Biotechnology, Shanghai, China). After centrifugation for 15 min at 14,000×*g*, supernatants were collected and incubated with the indicated antibodies followed by the addition of protein A/G beads (sc-2003, Santa Cruz, America). After incubation overnight at 4 °C, beads were washed four times with lysis buffer. Immunoprecipitated proteins were eluted by boiling with 4×SDS loading buffer.

### m^6^A-seq, transcriptome-seq, and information analysis

We performed the 2 × 150 bp paired-end sequencing (PE150) on an Illumina Novaseq™ 6000 (LC-Bio Technology CO., Ltd., Hangzhou, China) following the vendor’s recommended protocol. We used HISAT2 (http://daehwankimlab.github.io/hisat2/) to map reads to the reference genome. Mapped reads of IP and input libraries were provided for R package exomePeak (https://bioconductor.org/packages/exomePeak/), which identifies m^6^A peaks with a bed or bigwig format that can be adapted for visualization on the IGV software (http://www.igv.org/). MEME (http://meme-suite.org/) and HOMER (http://homer.ucsd.edu/homer/motif/) were used for de novo and known motif finding followed by localization of the motif concerning peak summit. Called peaks were annotated by intersection with gene architecture using R package ChIPseeker (https://bioconductor.org/packages/ChIPseeker/). Then StringTie (https://ccb.jhu.edu/software/stringtie/) was used to perform expression level for all mRNAs from input libraries by calculating FPKM (total exon fragments /mapped reads (millions) × exon length (kB)). The differentially expressed mRNAs were selected with log2 (fold change) >1 or log2 (fold change) <−1 and *p* value <0.05 by R package edgeR (https://bioconductor.org/packages/edgeR/).

### Mass spectrometric analysis

FLAG-NKAP Co-IP product and m^6^A Co-IP product were loaded on a 10% SDS- PAGE gel and stained with Coomassie brilliant blue. About 100 μL with 100 mM TEAB, 1 ug trypsin, and 1/300 CaCl_2_ were added into the gel, proteins were digested overnight at 37 °C. After gel digestion, the separated peptides were analyzed by Q Exactive^TM^ series mass spectrometer (Thermo Fisher), with ion source of Nanospray Flex™ (ESI), spray voltage of 2.1 kV, and ion transport capillary temperature of 320 °C. The resulting spectra were searched by the search engines: Proteome Discoverer 2.2 (PD 2.2, Thermo). Gene Ontology (GO) functional analysis was conducted using the InterProScan program against the non-redundant protein database (including Pfam, PRINTS, ProDom, SMART, ProSite, PANTHER), and the databases of COG (Clusters of Orthologous Groups) and KEGG (Kyoto Encyclopedia of Genes and Genomes) were used to analyze the protein family and pathway.

### JC-1 labeling

Mitochondrial membrane potential was measured by JC-1 (CBIC2, MCE, USA) according to Sivandzade’s protocol [35]. The fluorescence intensity ratio of red to green represented mitochondrial membrane potential. Treatment with 0.5 μM ferrostatin-1 or 5 μM alpha-tocopherol for 24 h.

### Statistical analysis

We used the random number method for random allocation. The sample size of each experiment was indicated in the corresponding figure legend. The sample size was determined by the SEM of our previous experiments. Other sample sizes were equal to, or more than the general sample size recommended in the previous reports. If the values obtained were more than twice the SEM of the mean, we excluded the samples. Statistical analyses were performed by SPSS version 22 software (SPSS Inc., Chicago, IL, USA) for experimental analyses and GraphPad Prism 6.0 (GraphPad Software, La Jolla, CA, USA) for statistical plots. All data were presented as mean ± standard deviation (SD) values. Statistical comparisons between two groups were carried out by Student’s *t*-test. One-way ANOVA was used for multiple-group comparisons. Survival curves were generated using the Kaplan–Meier method and compared using the log-rank test. The indicated *P* values (*P < 0.05 and **P < 0.01) were considered statistically significant.

## Supplementary information

Reproducibility checklist

Supplementary figure legends

Figure S1

Figure S2

Figure S3

Figure S4

Supplementary Table S1

Supplementary Table S2

## Data Availability

The datasets used and analyzed during the current study are available from the corresponding author.
