# Supplementary material for: RNA binding protein NKAP protects glioblastoma cells from ferroptosis by promoting SLC7A11 mRNA splicing in an m6A-dependent manner
Source: Cell Death Dis. 2022 Jan 21;13(1):73. doi: 10.1038/s41419-022-04524-2 (PMC8783023; doi:10.1038/s41419-022-04524-2)
Supplement: Supplementary file 2 — Supplementary figure legends [file 41419_2022_4524_MOESM2_ESM.docx]

**Supplementary figure legends**

**Figure S1.** Mitochondrial membrane potential of U87MG cells influenced by NKAP knockdown in the absence or presence of 0.5μM ferrostatin-1 or 5μM alpha-tocopherol for 24h measured by JC-1. Bars show mean ± SD of three independent experiments. *P < 0.05, **P < 0.01.

**Figure S2.** Relative mRNA expression of 44 significantly differentially expressed genes identified by ferroptosis qPCR array. All bars show mean ± SD of three independent experiments.

**Figure S3. SLC7A11 knockdown induced ferroptosis in U87MG cells.**

**A.** The interference effect of SLC7A11 knockdown in U87MG cell line. GAPDH was used as a loading control. **B.** Cell proliferation of U87MG cells influenced by SLC7A11 knockdown were examined using a CCK-8 assay. Cell growth was inhibited by SLC7A11 knockdown and partial reversed by 0.5μM ferrostatin-1 or 5μM alpha-tocopherol for 24h. **C.** Cell death induced by SLC7A11 knockdown in the absence or presence of 0.5μM ferrostatin-1 or 5μM alpha-tocopherol for 24h detected by flow cytometry in U87MG cell line. **D.** Lipid peroxidation level in U87MG cells influenced by SLC7A11 knockdown assessed by C11-BODIPY using flow cytometry immunolabelling in the absence or presence of 0.5μM ferrostatin-1 or 5μM alpha-tocopherol for 24h. All bars show mean ± SD of three independent experiments. *P < 0.05, **P < 0.01.

**Figure S4. Cycloleucine cannot induce cell death or increase lipid peroxidation level.**

**A.** Cell death in the absence or presence of 40μM cycloleucine for 24h detected by flow cytometry in U87MG cell line. **B.** Lipid peroxidation level in the absence or presence of 40μM cycloleucine for 24h assessed by C11-BODIPY using flow cytometry. All bars show mean ± SD of three independent experiments. *P < 0.05, **P < 0.01.
