# Supplementary figures and images for: RNA binding protein NKAP protects glioblastoma cells from ferroptosis by promoting SLC7A11 mRNA splicing in an m6A-dependent manner
Source: Cell Death Dis. 2022 Jan 21;13(1):73. doi: 10.1038/s41419-022-04524-2 (PMC8783023; doi:10.1038/s41419-022-04524-2)

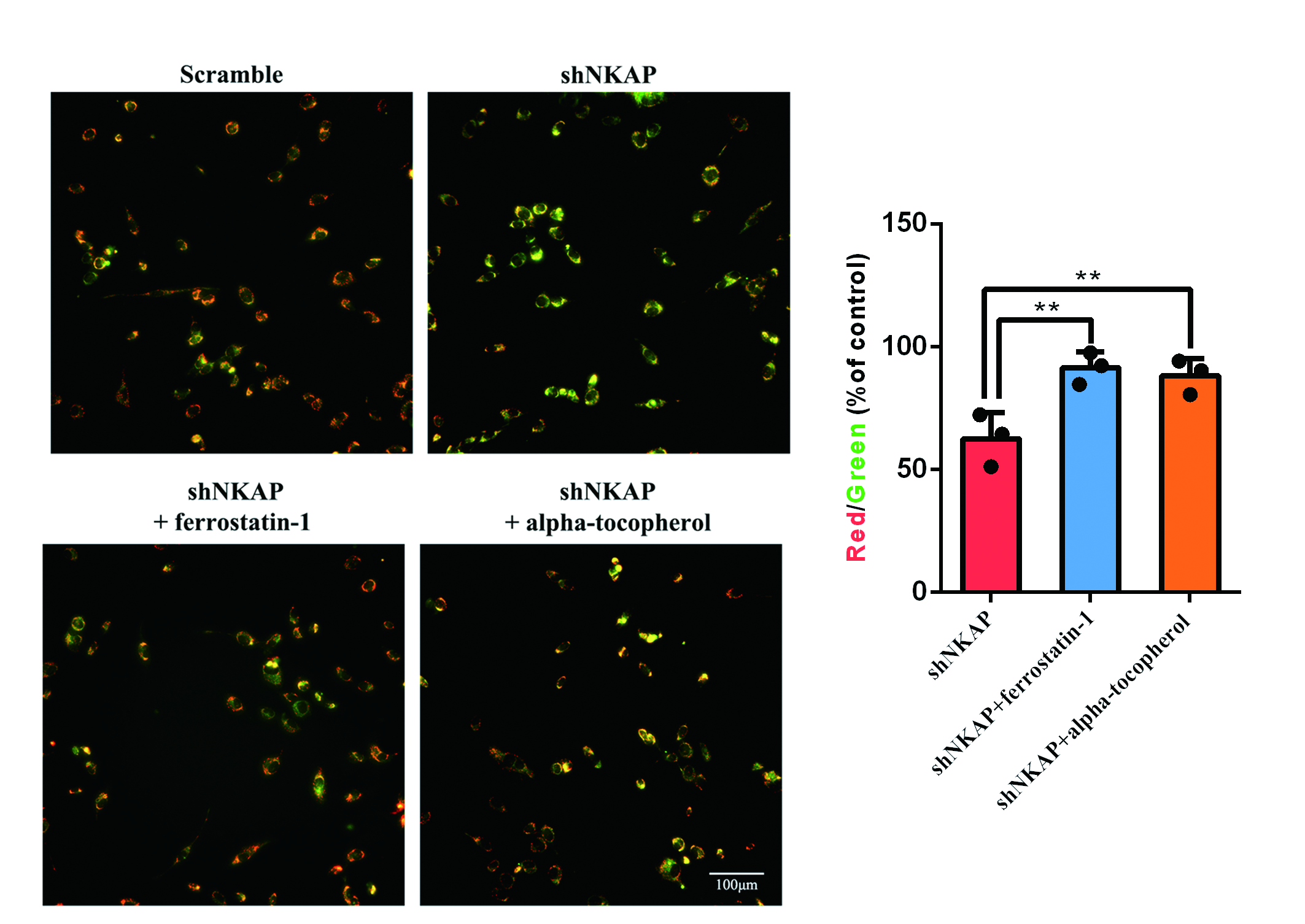

Supplement: Supplementary file 3 — Figure S1 [file 41419_2022_4524_MOESM3_ESM.tif]

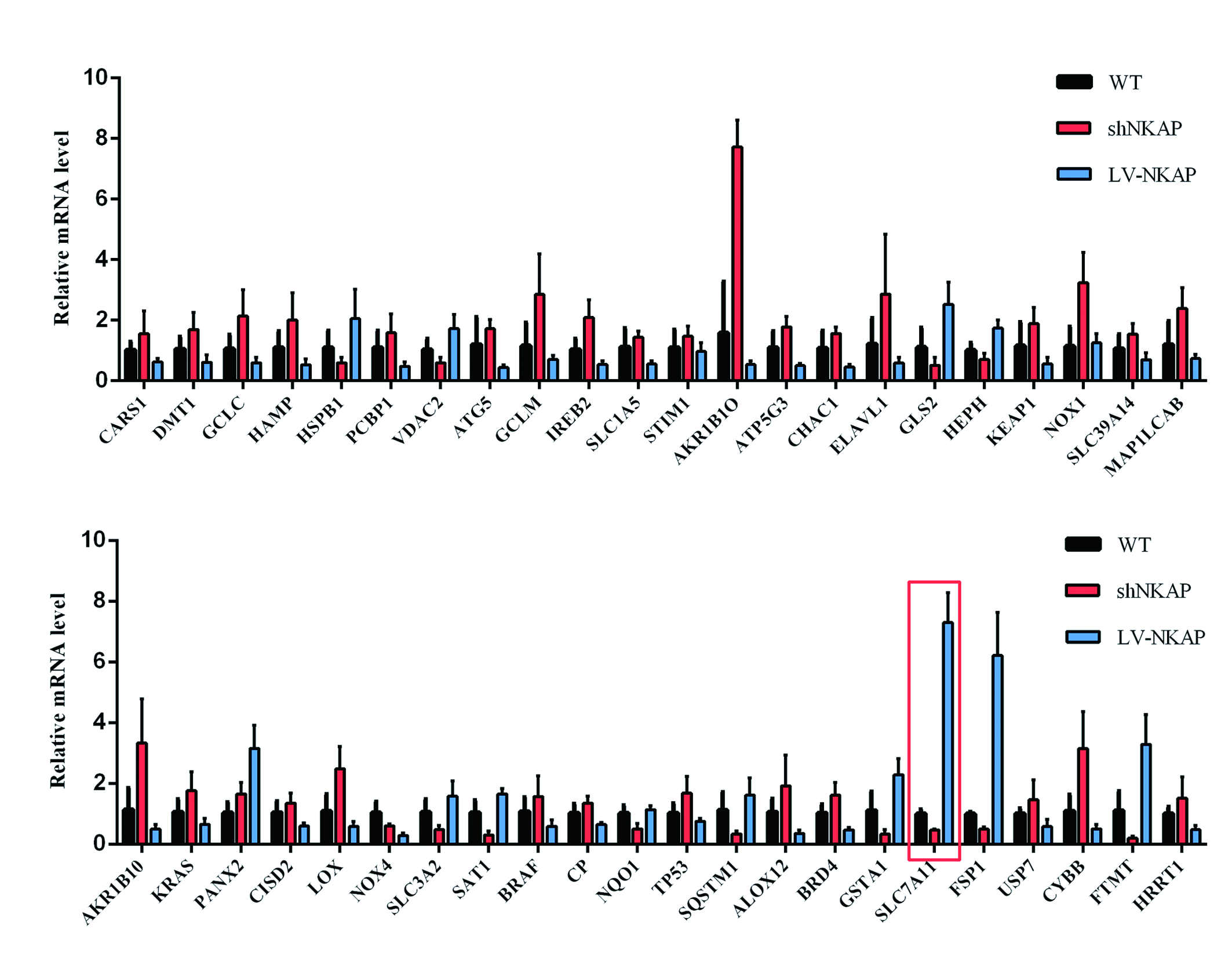

Supplement: Supplementary file 4 — Figure S2 [file 41419_2022_4524_MOESM4_ESM.tif]

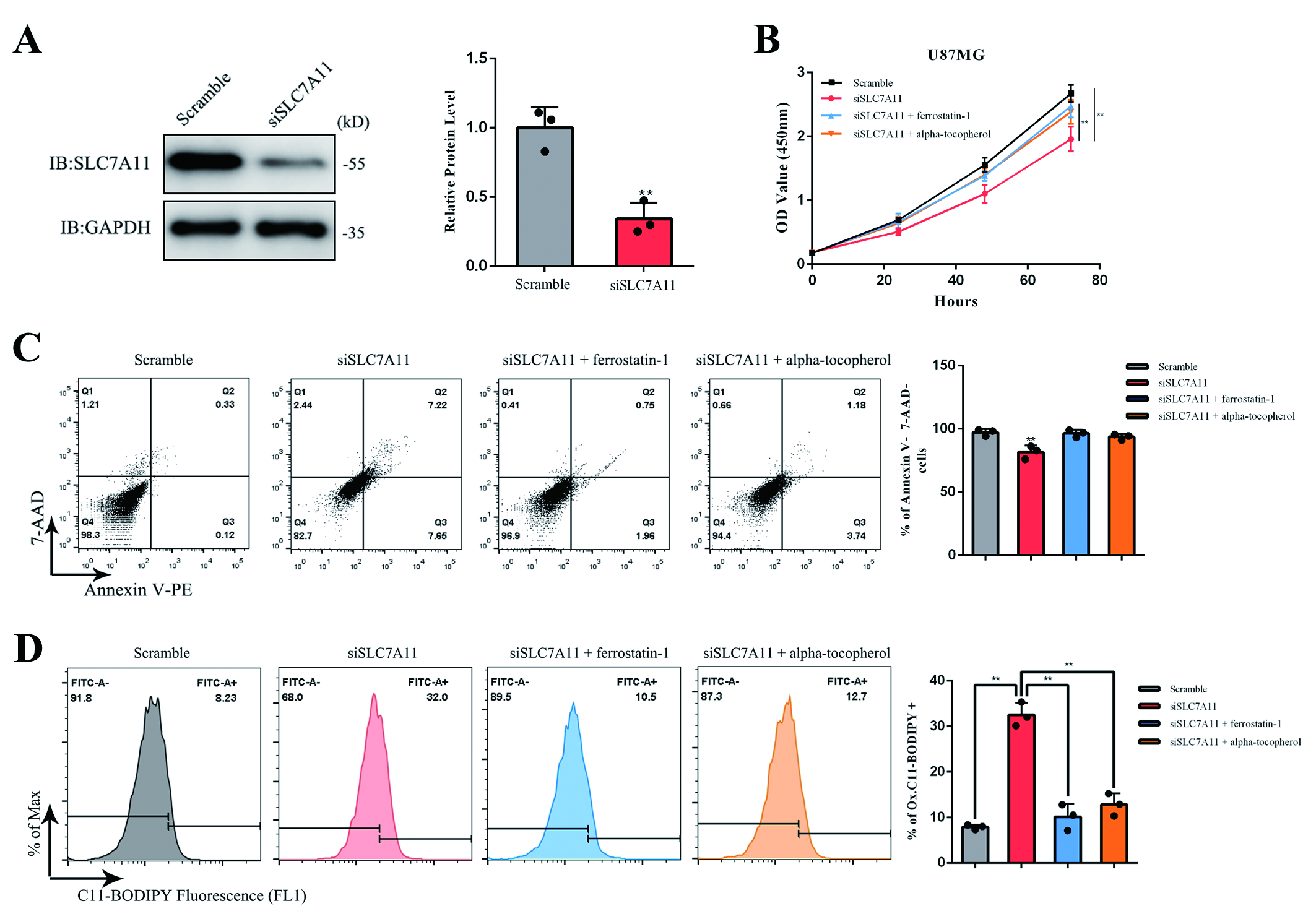

Supplement: Supplementary file 5 — Figure S3 [file 41419_2022_4524_MOESM5_ESM.tif]

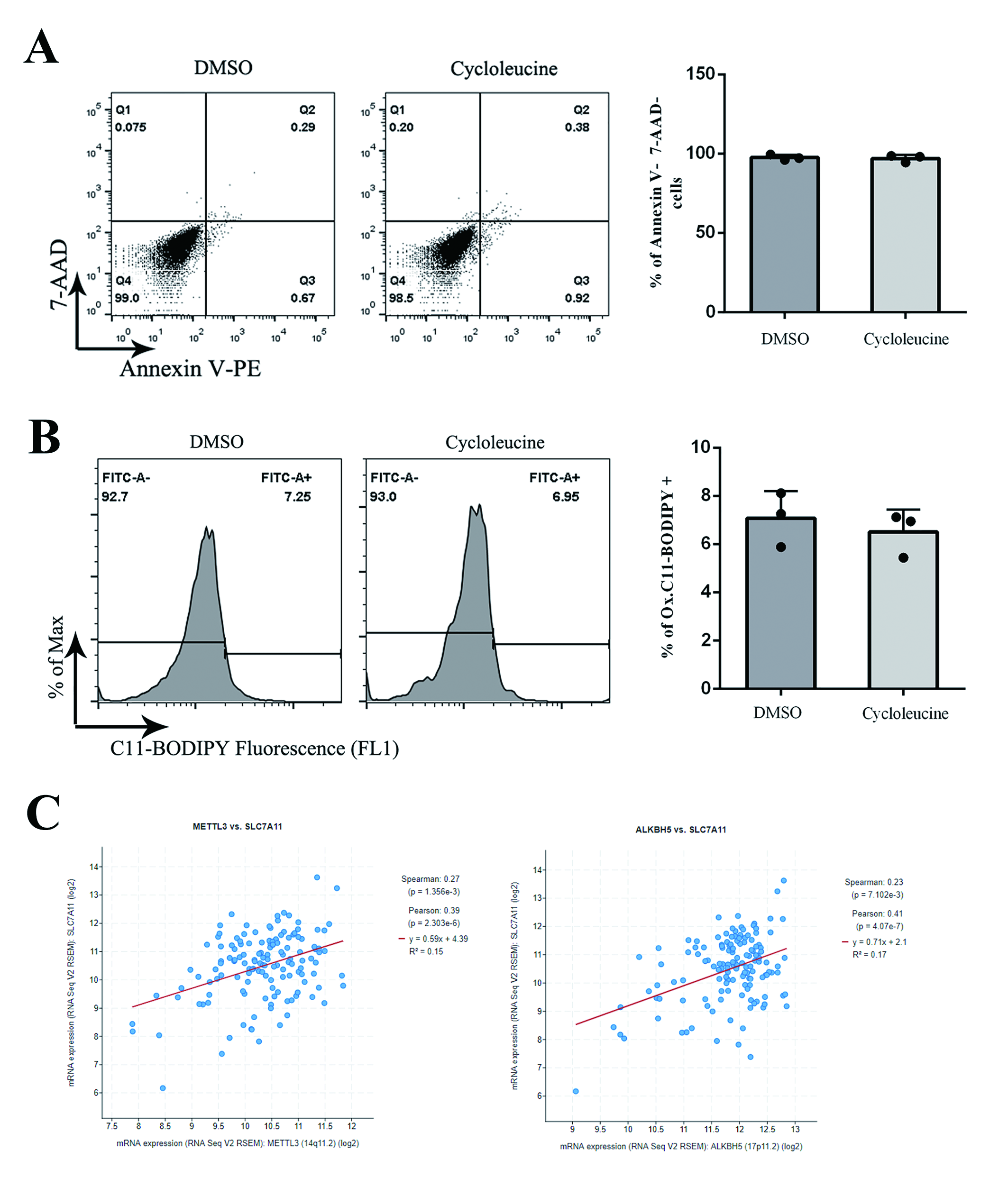

Supplement: Supplementary file 6 — Figure S4 [file 41419_2022_4524_MOESM6_ESM.tif]
