# Supplementary material for: RNA binding protein NKAP protects glioblastoma cells from ferroptosis by promoting SLC7A11 mRNA splicing in an m6A-dependent manner
Source: Cell Death Dis. 2022 Jan 21;13(1):73. doi: 10.1038/s41419-022-04524-2 (PMC8783023; doi:10.1038/s41419-022-04524-2)
Supplement: Supplementary file 7 — Supplementary Table S1 [file 41419_2022_4524_MOESM7_ESM.docx]

| **Supplementary Table S1. MS of NKAP-IP and m^6^A-IP screened out 17 overlapped proteins.** | | | |
| --- | --- | --- | --- |
| **Protein** | **Description** | **Gene** | **GO_NUM** |
| Q8VIJ6 | Splicing factor, proline- and glutamine-rich | Sfpq | 1 |
| F8VQC1 | Signal recognition particle subunit SRP72 | Srp72 | 4 |
| P32067 | Lupus La protein homolog | Ssb | 2 |
| P11031 | Activated RNA polymerase II transcriptional coactivator p15 | Sub1 | 2 |
| Q8BQ46 | TAF15 RNA polymerase II, TATA box binding protein (TBP)-associated factor | Taf15 | 2 |
| Q3TFA9 | Uncharacterized protein | Tmod3 | 2 |
| Q61033 | Lamina-associated polypeptide 2, isoforms alpha/zeta | Tmpo | 1 |
| Q04750 | DNA topoisomerase 1 | Top1 | 4 |
| P68372 | Tubulin beta-4B chain | Tubb4b | 1 |
| P26369 | Splicing factor U2AF 65 kDa subunit | U2af2 | 1 |
| Q9Z1Z0 | General vesicular transport factor p115 | Uso1 | 6 |
| Q3TFD9 | Vimentin | Vim | 2 |
| P62960 | Y-box-binding protein 1 | Ybx1 | 1 |
| Q921L4 | Histone H2B | LOC665622 | 1 |
| Q99MR8 | Methylcrotonoyl-CoA carboxylase subunit alpha, mitochondrial | Mccc1 | 1 |
| K7N7F1 | Myocyte-specific enhancer factor 2B | Mef2b | 2 |
| Q7TPV4 | Myb-binding protein 1A | Mybbp1a | 2 |
