# Supplementary material for: RNA binding protein NKAP protects glioblastoma cells from ferroptosis by promoting SLC7A11 mRNA splicing in an m6A-dependent manner
Source: Cell Death Dis. 2022 Jan 21;13(1):73. doi: 10.1038/s41419-022-04524-2 (PMC8783023; doi:10.1038/s41419-022-04524-2)
Supplement: Supplementary file 8 — Supplementary Table S2 [file 41419_2022_4524_MOESM8_ESM.docx]

| **Supplementary Table S2. Primers used in this study** | | |
| --- | --- | --- |
| **Genes** | **Primer sequence (5’-3’)** | |
| ***NKAP***  ***(Human)*** | F: GGCAAGCGTATCCCAAGAAGAG | R: TTCCGAAGTCGAACAGCCTCCA |
| ***SLC7A11***  ***(Human)*** | F: CTTTGTTGCCCTCTCCTGCTTC | R: CAGAGGAGTGTGCTTGTGGACA |
| ***GAPDH***  ***(Human)*** | F: GAACGGGAAGCTCACTGG | R: GCCTGCTTCACCACCTTCT |
| ***β-actin***  ***(Human)*** | F: AGGCCAACCGCGAGAAGATGACC | R: GAAGTCCAGGGCGACGTAGCAC |
| ***GPX4***  ***(Human)*** | F: CCGCCTTTGCCGCCTAC | R: TTTACTTCGGTCTTGCCTCACT |
| ***pre-mRNA***  ***SLC7A11***  ***(Human)*** | F: AGACCTCGTGAGCAGTTACCAG | R: AATACATCTTGATGCCTGTCCAG |
